# Comparison of type 2 diabetes prevalence estimates in Saudi Arabia from a validated Markov model against the International Diabetes Federation and other modelling studies

**DOI:** 10.1016/j.diabres.2013.12.036

**Published:** 2014-03

**Authors:** Abdulkareem J. Al-Quwaidhi, Mark S. Pearce, Eugene Sobngwi, Julia A. Critchley, Martin O’Flaherty

**Affiliations:** a Newcastle University, United Kingdom; b Ministry of Health, Saudi Arabia; c St. George's University of London, United Kingdom; d University of Liverpool, United Kingdom

**Keywords:** Modelling, Diabetes, Prevalence, Saudi Arabia

## Abstract

**Aims:**

To compare the estimates and projections of type 2 diabetes mellitus (T2DM) prevalence in Saudi Arabia from a validated Markov model against other modelling estimates, such as those produced by the International Diabetes Federation (IDF) Diabetes Atlas and the Global Burden of Disease (GBD) project.

**Methods:**

A discrete-state Markov model was developed and validated that integrates data on population, obesity and smoking prevalence trends in adult Saudis aged ≥25 years to estimate the trends in T2DM prevalence (annually from 1992 to 2022). The model was validated by comparing the age- and sex-specific prevalence estimates against a national survey conducted in 2005.

**Results:**

Prevalence estimates from this new Markov model were consistent with the 2005 national survey and very similar to the GBD study estimates. Prevalence in men and women in 2000 was estimated by the GBD model respectively at 17.5% and 17.7%, compared to 17.7% and 16.4% in this study. The IDF estimates of the total diabetes prevalence were considerably lower at 16.7% in 2011 and 20.8% in 2030, compared with 29.2% in 2011 and 44.1% in 2022 in this study.

**Conclusion:**

In contrast to other modelling studies, both the Saudi IMPACT Diabetes Forecast Model and the GBD model directly incorporated the trends in obesity prevalence and/or body mass index (BMI) to inform T2DM prevalence estimates. It appears that such a direct incorporation of obesity trends in modelling studies results in higher estimates of the future prevalence of T2DM, at least in countries where obesity has been rapidly increasing.

## 1 Introduction

T2DM is one of the most common non-communicable diseases in the world, and its levels are progressively increasing, particularly in developing countries [1]. It imposes a heavy burden on individuals and health care systems. The disease is associated with severe complications (e.g. blindness, lower limb amputations, and chronic renal failure) which affect health and productivity [2]. In 2011, diabetes caused around 4.6 million deaths globally in the 20–79 age group, and at least US$ 465 billion in healthcare expenditures, which was equivalent to 11% of total healthcare expenditures in adults [3]. Therefore, it is important for countries to have credible data on the trends in T2DM prevalence and its likely future projections. These data are required for proper health policy planning and resource allocation for the prevention and control of T2DM.

The Kingdom of Saudi Arabia (KSA) is one of the largest and wealthiest countries in the region of Middle East and North Africa (MENA). It is a leading oil-producing country, and has witnessed massive socioeconomic developments in the past five decades with rapid urbanisation and changes in the population lifestyles. KSA is now classified by the IDF to be among the top 10 countries globally with the highest projected prevalence of diabetes in 2011 (16.2%) and 2030 (20.8%) [1]. Furthermore, the prevalence of some risk factors for T2DM in KSA (e.g. obesity) has also been estimated to be among the highest in the world [4].

Epidemiological modelling is a valuable tool for estimation and future prediction of T2DM prevalence. The IDF [1] and other international modelling studies [5–9] have generated estimates of the prevalence of diabetes in different countries of the world, including KSA, at different time points. However, for KSA, most of such estimates appear to noticeably underestimate the true situation, as they have been well surpassed by the local ‘observed’ data.

We developed the ‘Saudi IMPACT Diabetes Forecast Model’ to estimate the trends and likely future projections of T2DM in KSA, based primarily on the trends in the prevalence of obesity and smoking as risk factors. Obesity has been well recognised as one of the most important risk factors for T2DM [10]. In addition, smoking is not only a reflection of unhealthy lifestyle (with low physical activity and unhealthy diet), but it has also been recognised as an independent risk factor for T2DM in several large prospective studies that have adjusted their results for many potential covariates (e.g. obesity, physical activity, age, etc.) [11]. We validated our model against local observed data from the STEPS (STEPwise approach to non-communicable diseases Surveillance) survey [12] in 2005. This study aims to provide a detailed comparison of the estimates of T2DM prevalence in KSA by the Saudi IMPACT Diabetes Forecast Model against the estimates of the IDF Diabetes Atlas [1] and the GBD study [5].

## 2 Methods

### 2.1 The model

The Saudi IMPACT Diabetes Forecast Model is a discrete-state Markov model, implemented in Microsoft Excel spreadsheets. It was originally designed for the MedCHAMPS project [13,14] for use in middle and low income countries, where extensive data on T2DM risk factors, prevalence, and complications may not be available. It integrates information on the trends in the adult Saudi population structure by age group and sex (obtained from the Central Department of Statistics and Information (CDSI) [15] and the United Nations (UN) population estimates [16]), and the trends in the prevalence of two risk factors for T2DM: obesity (BMI ≥ 30 kg/m^2^) and current active smoking (obtained from local population-based surveys). The model estimates the trends in prevalence of T2DM in the Saudi adults aged ≥25 years during the 30-year period of 1992–2022. The age- and sex-specific prevalence of T2DM in the starting year of modelling (1992) was obtained from a nationwide population-based study, which used the WHO 1985 diagnostic criteria and oral glucose tolerance test (OGTT) for diagnosis of T2DM [17].

**Fig. 1:**
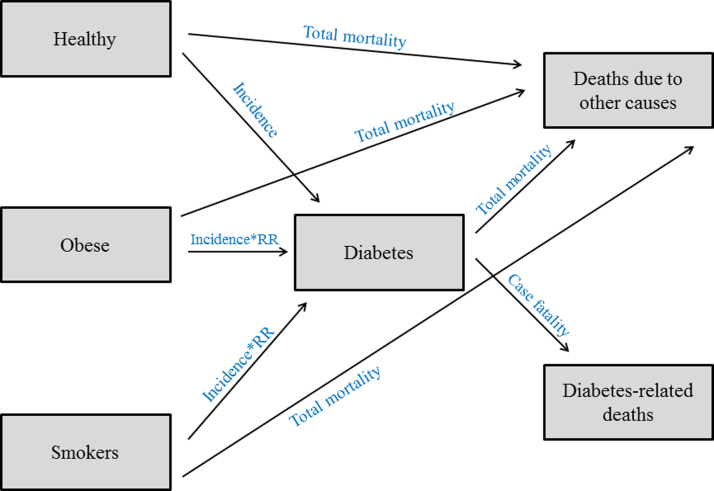
Simple illustration of the structure of the Saudi IMPACT Diabetes Forecast Model.

Fig. 1 shows a simple illustration of the model structure. The model assumes that the population is divided into three discrete pools (health states): those who are obese, those who are smokers, and those who are ‘healthy’ (i.e. non-obese, non-smokers, and not having T2DM). Individuals can make transitions from the three main health states or remain in the same state during each modelling cycle (one year). Individuals can make transitions to the (Diabetes) state (i.e. they develop T2DM), or die due to other causes. Individuals in the (Diabetes) state can die as a result of T2DM or due to other causes. Individuals with T2DM cannot make transition back to the three main states, assuming a zero remission rate.

**Fig. 2:**
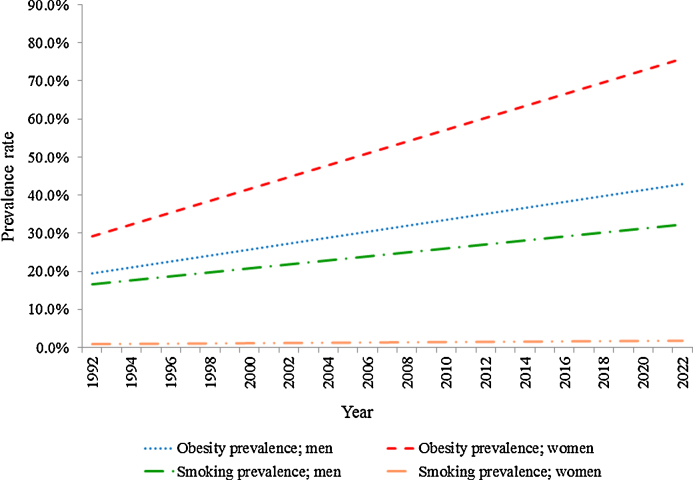
Trends in prevalence of obesity and smoking in the adult Saudi men and women (1992–2022).

The size of the (Obese) and (Smokers) states were determined by the age- and sex-specific prevalence of obesity and active smoking, as obtained from nationally-representative local surveys. Three surveys were used to obtain the prevalence of obesity in 1992 [17], 1997 [18], and 2005 [12]. In addition, two surveys were used to derive the prevalence of active smoking in 1992 [19] and 2005 [12]. Data for missing years were estimated through linear interpolation, while data for future trends were estimated through linear extrapolation, assuming similar rates of increase as that observed from surveys. Fig. 2 demonstrates the trends in prevalence of obesity and smoking in the adult Saudi men and women over the modelling period (1992–2022), assuming a linear increasing trends.

The potential overlaps between the model health states were handled in three different ways. First, smoking prevalence was multiplied by obesity prevalence in order to estimate the proportion of population who were both obese and smokers. Then, such a proportion was subtracted from the ‘original’ smoking prevalence, to leave in the (Smokers) state only those individuals who were smokers but not obese. Second, we estimated the number of individuals with T2DM (in the Diabetes state) in whom the disease was assumed to be ‘caused’ by obesity as an exposure, through multiplying the ‘*population attributable risk*’ [20] by the size of (Diabetes) state. Then, the number of such individuals was subtracted from the total obese individuals in population, to leave in the (Obese) state only those obese individuals who do not have T2DM. Finally, we applied the same previous approach of the *population attributable risk*, to leave in the (Smokers) states only those people who are smokers, but not having T2DM.

The transition from (Healthy) to (Diabetes) states is informed by the *incidence of T2DM* (incidence in ‘healthy’ people only), while the transition from (Obese) to (Diabetes) states is informed by the *incidence of T2DM* × *relative risk (RR) of diabetes in obese individuals*, and the transition from (Smokers) to (Diabetes) states is informed by the *incidence of T2DM* × *RR of diabetes in smokers*. Moreover, the transitions from any of the three main states (Healthy, Obese, and Smokers) to the state of (Deaths due to other causes) are informed by the *total mortality rate*. On the other hand, transition from the (Diabetes) state to the state of (Diabetes-related deaths) is informed by the *case fatality rate*.

The model does not show explicit transitions from the (Healthy) state to (Obese) or (Smokers), because of lack of data needed to inform such transitions. In addition, as a simplifying assumption, the prevalence of overweight (BMI 25–29.9 kg/m^2^) is not explicitly modelled, and we only considered the trends in obesity prevalence.

The age- and sex-specific incidence and case fatality rate of T2DM, in addition to the total mortality rate of population were estimated using DISMOD 2 [21], which is a validated generic disease model designed to supplement data on a disease epidemiology by exploiting the causal relations between the various available parameters. DISMOD 2 provides ‘internally-consistent’ estimates of diabetes incidence, case fatality rate, and total mortality rate, based on a set of differential equations that describe the disease process [21]. DISMOD 2 has been widely used by the WHO's Global Burden of Disease (GBD) Study and other modelling studies. DISMOD 2 provides an estimated incidence rate for the overall population (i.e. for obese, smokers, non-obese and non-smokers). However, assuming that the overall incidence of diabetes is a weighted sum of the incidence among exposed (Obese and Smokers) and non-exposed (Healthy) [22], we calculated the incidence rate of diabetes among (Healthy) people only to inform the transition between the (Healthy) state and (Diabetes) state. We then adjusted this incidence rate by applying the RRs of diabetes in obese and in smokers in order to inform the transitions between the (Obese) and (Smokers) states to (Diabetes) state.

The RR of diabetes in obese and in smoker individuals were obtained from two recent systematic reviews and meta-analyses [10,11]. Table 1 shows the age- and sex-specific parameters used to set the transition probabilities in the model.

We conducted two main methods of sensitivity analyses in order to test the potential uncertainties around the model parameters. First, we used the ‘analysis of extremes’ method [23,24], where all model parameters (except population structure) were conservatively set at 20% higher and 20% lower values than the base-case scenario. The model was run with this distribution of extreme values and the uncertainty intervals (UIs) were estimated accordingly. Second, we used the ‘scenario analysis’ method, where two reasonable scenarios were assumed for the projected obesity prevalence. For *scenario 1*, we assumed that the obesity trends would continue to increase at the same annual rate as that observed from the national surveys. In *scenario 2*, we assumed that the projected obesity trends would be ‘capped’ at the highest ‘observed’ value in any age group for each sex separately. The highest observed value for obesity prevalence in men was 34.5% in those aged 35–44 years, while in women was 58.8% in the age group 45–54 years. Therefore, the capping point was assumed to be 35% in men and 60% in women.

### 2.2 Comparison with other models

We compared our model estimates with those recently published by two independent modelling exercises, which generated estimates and projections of T2DM prevalence in a large number of countries around the world, including KSA.

#### 2.2.1 The IDF Diabetes Atlas, fifth edition [1,25]

The IDF used logistic regression to model diabetes prevalence rates in KSA, which were derived from five national population-based surveys. The smoothed age- and sex-specific prevalences were then applied to the national population distribution for the years 2011 and 2030 (using the UN population estimates and the world population distribution) to estimate national prevalence of diabetes. The IDF methodology used changes in age, sex and urbanisation as covariates for estimating diabetes prevalence.

#### 2.2.2 The GBD model [5]

The GBD study (Global Burden of Metabolic Risk Factors of Chronic Diseases Collaborating Group (Blood Glucose)) used three national population-based surveys of T2DM prevalence in KSA [12,17,26] and used a multi-level statistical approach (Bayesian hierarchical modelling) to model the trends in fasting plasma glucose (FPG) and T2DM prevalence in adults aged ≥25 years. The estimates were informed by several country-level covariates. These covariates were national income (natural logarithm of the per-head gross domestic product), urbanisation (proportion of population that lived in urban areas), age-standardised mean BMI (from a previous GBD systematic analysis of country data [4]), and national availability of multiple food types for human consumption (from the food balance sheets of the Food and Agriculture Organisation (FAO) of the UN). The presented estimates were age standardised to the WHO reference population.

## 3 Results

The prevalence of T2DM among the Saudi population aged ≥25 years is estimated to rise substantially from 8.5% (UI: 6.8–10.2%) in 1992 to 31.4% (UI: 25.5–37.0%) in 2013 and 44.1% (UI: 35.4–52.5%) by 2022, assuming that the observed prevalence rates of both smoking and obesity will continue to increase. In comparison, the prevalence of T2DM is estimated to increase to 30.8% (UI: 25.2–36.2%) in 2013 and 39.5% (UI: 32.5–45.9%) by 2022, assuming capped obesity trends at 35% in men and 60% in women. The estimated number of people with T2DM in KSA will increase substantially from around 555,000 in 1992 to approximately 7.4 million by 2022, assuming a continuing increase of obesity levels, and nearly 6.6 million, assuming capped obesity levels.

We compared the estimates of T2DM prevalence in KSA from the Saudi IMPACT Diabetes Forecast Model against the estimates of the IDF Diabetes Atlas (fifth edition) and the GBD Study (Table 2).

### 3.1 Comparison with the IDF Diabetes Atlas, fifth edition [1,25]

The Saudi IMPACT Diabetes Forecast Model estimated the total T2DM prevalence in the Kingdom (in adults aged ≥25 years) at 29.2% in 2011 and 44.1% in 2022 (assuming a continuing linear increase in obesity trends “scenario 1”), or at 28.9% in 2011 and 39.5% (assuming capping of future obesity trends “scenario 2”). In comparison, the IDF has estimated the prevalence of diabetes in KSA in adults aged 20–79 years at 16.2% in 2011 and 20.8% in 2030.

### 3.2 Comparison with the GBD estimates [5]

The Saudi IMPACT Diabetes Forecast Model estimated the prevalence of diabetes in KSA among adult men and women respectively as follows: 17.7% and 16.4% in 2000, and 26.7% and 24.7% in 2008. The model results for 2000 and 2008 were the same with assuming a continuing linear increase (scenario 1), or capping (scenario 2) of obesity trends. On the other hand, the estimates of the GBD model for men and women respectively were 17.5% and 17.7% in 2000, and 22.0% and 21.7% in 2008.

## 4 Discussion

We developed and validate the Saudi IMPACT Diabetes Forecast Model, which is a Markov model with relatively few data input requirements. The model might be most suitable for use in less developed settings with limited data on T2DM. In addition to the demographic trends, the model also used the trends in two risk factors for T2DM in KSA, as well as literature-derived transition hazards. This study presents a comparison between the results from this model against that of the IDF Diabetes Atlas (fifth edition) [1] and the GBD study [5].

In general, reliability and accuracy of T2DM prevalence estimates are highly dependent on the data sources used in modelling process and the model structure and methodology. Thus, comparing the results of different T2DM prevalence estimates may be difficult, as different models often utilise different data sources, apply different methodologies for estimation and projections, and use different assumptions. Furthermore, Danaei et al. [5] reviewed the available global diabetes estimates and reported some other potential reasons for variations in their results. For instance, the definition of diabetes varied in different studies, as diagnostic criteria are repeatedly changeable over time. In addition, studies were different in their populations of interest, and some of them used data sources with subnational samples, regarding them as equally representative of national populations. This could lead to biased results, as those specific subnational groups might differ from the general populations in many aspects such as prevalence of some risk factors for diabetes.

This study revealed that the Saudi IMPACT Diabetes Forecast Model resulted in significantly higher estimates and projections of T2DM prevalence in KSA than that estimated by the IDF for 2011 and 2030 [1]. These considerable differences between the two studies could be mainly attributed to the different methods of modelling of T2DM prevalence and the covariates used for that purpose. According to the IDF, the estimation approach was deliberately kept simple and conservative. The IDF model used logistic regression method and based its predictions for 2030 on predicted demographic changes (urbanisation and ageing). Moreover, the IDF model did not attempt to directly account for the effects of changes in T2DM risk factors (e.g. obesity). The IDF has acknowledged this as a limitation which was likely to result in underestimation of T2DM prevalence if the levels of obesity and other risk factors continue to rise [1].

Also, several previous modelling studies [6–9] resulted in lower estimates and projections of diabetes prevalence in KSA at different time points than the Saudi IMPACT Diabetes Forecast Model (Table 3). Shaw et al. [6] and Wild et al. [7] used local population-based surveys (also used by the IDF and GBD models) as data sources of diabetes prevalence in KSA. On the other hand, the oldest two studies (King et al. [8] and Amos et al. [9]) used old data from Oman [27] (a neighbouring country to KSA with similar socioeconomic characteristics) and extrapolated such data to KSA. In general, all these four modelling studies used only demographic changes and level of urbanisation as informants of diabetes prevalence estimates and projections.

On the contrary, the Saudi IMPACT Diabetes Forecast Model utilised a different estimation approach (Markov modelling). It used only the prevalence of T2DM for the starting year (1992) along with the demographic trends of the Saudi population (1992–2022) and the trends in prevalence of two risk factors (obesity and smoking) over the same 30-year-period. In addition, the model used a number of transition parameters, such as the estimated incidence of T2DM, case fatality rate, general mortality and evidence-based RRs. The Saudi IMPACT Diabetes Forecast Model used published nationally representative, population-based studies to obtain data on the prevalence of obesity and smoking in KSA, considering two reasonable scenarios of the future obesity trends.

The results of the GBD model [5] and the Saudi IMPACT Diabetes Forecast Model are very comparable, in spite of differences in the general methods of estimation. This similarity in results of the two models could be attributed primarily to the several covariates used in both models to estimate T2DM prevalence. In contrast to the other models which used only ageing and level of urbanisation as covariates, the GBD model incorporated more covariates to inform its estimates. However, among all these covariates, mean BMI is likely to have the most important contribution to the higher estimates of the GBD model than that of the IDF model; because mean BMI could serve as a ‘direct’ informant of the trends in obesity levels. Another GBD modelling study for trends in the global BMI [4] showed that the estimated mean BMI in Saudi Arabia followed a linear increase between 1980 and 2008. The estimates of mean BMI (kg/m^2^) in men and women in Saudi Arabia were respectively as follows: 25.0 (uncertainty interval (UI): 23.8–26.3) and 26.3 (UI: 24.8–27.8) in 1980, 25.9 (UI: 25.6–26.2) and 27.3 (UI: 26.9–27.8) in 1990, 27.0 (UI: 26.6–27.4) and 28.5 (UI: 28.0–29.0) in 2000, and 27.9 (UI: 27.2–28.6) and 29.6 (UI: 28.7–30.5) in 2008. Furthermore, the GBD estimates showed that the region of ‘North Africa and Middle East’ has witnessed the largest increase in mean BMI in men and women between 1980 and 2008 after the region of ‘Oceania’. Saudi Arabia was among the top countries with the highest increase in mean BMI within its region [4]. Country-specific estimates of the prevalence of obesity (BMI ≥ 30 kg/m^2^) were not reported. However, the region of ‘North Africa and Middle East’ had the seventh (among the 21 GBD regions of the world) highest prevalence of obesity in men, and the second highest in women between 1980 and 2008. In men, the estimated prevalence of obesity in that region increased substantially from <10% in 1980 to 20–30% in 2008. On the other hand, obesity prevalence in women increased from 10–20% in 1980 to 30–40% in 2008 [4]. Taking that into account, the trends in obesity prevalence seems then to be a better option when modelling the future burden of T2DM. The Association of Public Health Observatories (APHO) Diabetes Prevalence Model [28] is a recent consistent example that reported similar variations in the estimated prevalence of diabetes when compared to other models. The APHO Diabetes Prevalence Model used the trends in overweight and obesity in England to estimate diabetes prevalence, which was approximately one third higher than that estimated by the IDF for 2010.

The Saudi IMPACT Diabetes Forecast Model offers to the health policy planners in KSA robust and validated estimates and projections of the burden of T2DM, and shows that the currently high levels of the disease are expected to continue increasing during the next decade, even assuming some degree of levelling off of obesity trends. Therefore, there is an urgent need for applying effective, aggressive and multisectoral prevention measures to promote healthy diet, physical activity, and smoking cessation. It is recommended to improve public awareness to increase consumption of fruit and vegetables, restrict consumption of fat and sugar, practice a regular physical activity, and quit tobacco smoking [29,30]. It is also important to establish (or maintain) relevant legislation to enforce food industry to make clear and informative food labelling, to incorporate physical education into curriculums at girls schools, to strictly prohibit smoking at public and work places, and to increase tobacco prices and ban its selling to children and adolescents [29,30]. Beside the primary prevention measures which aim to reduce the prevalence of T2DM, it is also recommended to implement mass screening programmes to diagnose those people with ‘occult’ disease for early treatment and prevention of disease progression [29,30].

## 5 Conclusion

The Saudi IMPACT Diabetes Forecast Model and the GBD model produced comparable estimates of T2DM prevalence in Saudi Arabia, as both models directly incorporated the trends in obesity prevalence and/or BMI to inform the estimates. In contrast, the IDF and other modelling studies relied solely on demographic trends and urbanisation as covariates, and produced lower estimates of T2DM prevalence in Saudi Arabia. Hence, it appears that the direct incorporation of obesity trends (as a strong predictor of T2DM) in modelling studies results in higher estimates of the future prevalence of T2DM, particularly in countries and regions with high levels of and increasing trends in obesity prevalence.

## Conflict of interest statement

None.

## Figures and Tables

**Table 1:** Transition hazards used in the Saudi IMPACT Diabetes Forecast Model.

Transition parameter	Data source	Men – age group (years)						Women – age group (years)					
		25–34	35–44	45–54	55–64	65–74	75+	25–34	35–44	45–54	55–64	65–74	75+
Estimated incidence rate of diabetes/1000 population	DISMOD 2	12.90	17.70	18.90	20.70	22.40	26.70	12.90	15.00	15.90	16.70	19.70	30.70
Estimated case fatality rate (%)	DISMOD 2	0.15	0.39	0.67	1.20	1.35	2.10	0.16	0.43	0.62	0.96	1.90	4.62
Estimated total mortality rate/1000 population	DISMOD 2	0.10	0.50	1.10	2.50	3.30	6.10	0.10	0.50	1.00	1.80	4.10	11.60

RR of diabetes if obese	Guh et al. [10]	6.74						12.41					
RR of diabetes if a smoker	Willi et al. [11]	1.44						1.44					

**Table 2:** Comparison of the Saudi IMPACT T2DM Forecast Model against the IDF (2011) model and the GBD (2011) model.

	IDF (2011) [1,25]		GBD (2011) [5]		Saudi IMPACT Diabetes Forecast Model	
Estimated DM prevalence in Saudi Arabia (%)	2011	Total: 16.2	2000	Males: 17.5 Females: 17.7	2000	Males: 17.7 Females: 16.4 Total: 17.2
	2030	Total: 20.8	2008	Males: 22.0 Females: 21.7	2008	Males: 26.7 Females: 24.7 Total: 25.9
					2011	Males: 29.8 Females: 28.1 Total: 29.2
					2022	Males: 41.3 Females: 47.7 Total: 44.1

Age of study population (years)	20–79		25+		25+	
						

Main data sources for DM prevalence in Saudi Arabia	Al-Nuaim et al. [31] El-Hazmi et al. [32] Warsy and El-Hazmi [17] Al-Nozha et al. [26] WHO STEPS [12]		Warsy and El-Hazmi [17] Al-Nozha et al. [26] WHO STEPS [12]		Warsy and El-Hazmi [17] (for starting year prevalence) WHO STEPS [12] (for validation)	
						

Estimation methodology	Logistic regression modelling		Complex multi-level Bayesian hierarchical modelling		Markov modelling	
						

Covariates used for estimating DM prevalence	• Urbanisation • Ageing		• National income • Urbanisation • National availability of multiple food types • Age-standardised mean BMI		• Trends in population structure • Trends in obesity prevalence • Trends in smoking prevalence • Estimated incidence of T2DM • Estimated case-fatality rate • Evidence-based estimates of RRs for transition probabilities	

**Table 3:** Comparison of the Saudi IMPACT T2DM Forecast Model against other modelling studies.

	Shaw et al. [6]		Wild et al. [7]		King et al. [8]		Amos et al. [9]		Saudi IMPACT Diabetes Forecast Model	
Estimated DM prevalence in Saudi Arabia (%)	2010	Total: 13.6	2000	Total: 6.2	1995	Total: 8.7	1995	Total: 10.0	1995	Total: 11.1
	2030	Total: 17.0	2030	Total: 8.1	2000	Total: 9.1	2000	Total: 12.0	2000	Total: 17.2
					2025	Total: 10.1	2010	Total: 13.8	2010	Total: 28.1
									2022	Total: 44.1

Age of study population (years)	20–79		20+		20+		20+		25+	

Main data sources for DM prevalence in Saudi Arabia	Al-Nuaim et al. [31] El-Hazmi et al. [32] Al-Nozha et al. [26]		El-Hazmi et al. [32]		Asfour et al. [27]*(Study from Oman)*		El-Hazmi et al. [33] Asfour et al. [27]*(study from Oman)*		Warsy and El-Hazmi [17] (for starting year prevalence) WHO STEPS [12] (for validation)	

Estimation methodology	Logistic regression modelling		DISMOD 2		Age-specific diabetes prevalence estimates were applied to UN population estimates and projections		Country-specific diabetes prevalence data were applied to the corresponding national age distribution		Markov modelling	

Covariates used for estimating DM prevalence	• Demographic changes • Urbanisation		• Demographic changes • Urbanisation		• Trends in population size and age structure • Urbanisation		• Level of economic development (GNP per capita) • Urbanisation		• Trends in population structure • Trends in obesity prevalence • Trends in smoking prevalence • Estimated incidence of T2DM • Estimated case-fatality rate • Evidence-based estimates of RRs for transition probabilities	

## References

[1] Whiting D.R., Guariguata L., Weil C., Shaw J. (2011). IDF Diabetes Atlas: global estimates of the prevalence of diabetes for 2011 and 2030. Diabetes Res Clin Pract.

[2] Ekoe J.-M., Zimmet P., Ekoe J.-M., Zimmet P., Williams R. (2001). Diabetes mellitus: diagnosis and classification. The epidemiology of diabetes mellitus: an international perspective.

[3] IDF Diabetes Atlas (2011). 5th edition: International Diabetes Federation. http://www.idf.org/diabetesatlas/5e/.

[4] Finucane M.M., Stevens G.A., Cowan M.J., Danaei G., Lin J.K., Paciorek C.J. (2011). National, regional, and global trends in body-mass index since 1980: systematic analysis of health examination surveys and epidemiological studies with 960 country-years and 9· 1 million participants. Lancet.

[5] Danaei G., Finucane M.M., Lu Y., Singh G.M., Cowan M.J., Paciorek C.J. (2011). National, regional, and global trends in fasting plasma glucose and diabetes prevalence since 1980: systematic analysis of health examination surveys and epidemiological studies with 370 country-years and 2.7 million participants. Lancet.

[6] Shaw J.E., Sicree R.A., Zimmet P.Z. (2010). Global estimates of the prevalence of diabetes for 2010 and 2030. Diabetes Res Clin Pract.

[7] Wild S., Roglic G., Green A., Sicree R., King H. (2004). Global prevalence of diabetes: estimates for the year 2000 and projections for 2030. Diabetes Care.

[8] King H., Aubert R.E., Herman W.H. (1998). Global burden of diabetes, 1995–2025: prevalence, numerical estimates, and projections. Diabetes Care.

[9] Amos A.F., McCarty D.J., Zimmet P. (1997). The rising global burden of diabetes and its complications: estimates and projections to the year 2010. Diabet Med.

[10] Guh D., Zhang W., Bansback N., Amarsi Z., Birmingham C.L., Anis A. (2009). The incidence of co-morbidities related to obesity and overweight: a systematic review and meta-analysis. BMC Public Health.

[11] Willi C., Bodenmann P., Ghali W.A., Faris P.D., Cornuz J. (2007). Active smoking and the risk of type 2 diabetes: a systematic review and meta-analysis. JAMA.

[12] WHO STEPwise Approach to NCD Surveillance (2005). Country-Specific Standard Report, Saudi Arabia. http://www.who.int/chp/steps/2005_SaudiArabia_STEPS_Report_EN.pdf.

[13] Bowman S., Unwin N., Critchley J., Capewell S., Husseini A., Maziak W. (2012). Use of evidence to support healthy public policy: a policy effectiveness–feasibility loop. WHO Bull.

[14] Maziak W., Critchley J.A., Zaman S., Unwin N., Capewell S., Bennett K., Unal B., Husseini A., Ben Romdhane H., Phillimore P., MedCHAMPS collaboration (2013, Aug). MEDiterranean studies of Cardiovascular disease and Hyperglycaemia: Analytical Modelling of Population Socio-economic transitions (MedCHAMPS): Rationale and Methods. Int J Public Health.

[15] Central Department of Statistics and Information (2013). Kingdom of Saudi Arabia (Official website). http://www.cdsi.gov.sa/english/.

[16] The United Nations; Department of Economic and Social Affairs (2011). Population Division, Population Estimates and Projections Section. Detailed indicators. http://esa.un.org/unpd/wpp/unpp/panel_indicators.htm.

[17] Warsy A.S., El-Hazmi M.A.F. (1999). Diabetes mellitus, hypertension and obesity—common multifactorial disorders in Saudis. East Mediterr Health J.

[18] Al-Nozha M.M., Al-Mazrou Y.Y., Al-Maatouq M.A., Arafah M.R., Khalil M.Z., Khan N.B. (2005). Obesity in Saudi Arabia. Saudi Med J.

[19] Jarallah J.S., Al-Rubeaan K.A., Al-Nuaim A.R.A., Al-Ruhaily A.A., Kalantan K.A. (1999). Prevalence and determinants of smoking in three regions of Saudi Arabia. Tob Control.

[20] Hennekens C.H., Buring J.E., Mayrent S.L. (1987). Epidemiology in medicine.

[21] Barendregt J.J., Oortmarssen G.J.v., Vos T., Murray C.J. (2003). A generic model for the assessment of disease epidemiology: the computational basis of DisMod II. Popul Health Metrics.

[22] Szklo M., Nieto F.J. (2007). Epidemiology: beyond the basics. Second edition.

[23] Walker D., Fox-Rushby J. (2001). Allowing for uncertainty in economic evaluations: qualitative sensitivity analysis. Health Policy Plan.

[24] Briggs A. (2005). Probabilistic analysis of cost-effectiveness models: statistical representation of parameter uncertainty. Value Health.

[25] Guariguata L., Whiting D., Weil C., Unwin N. (2011). The International Diabetes Federation diabetes atlas methodology for estimating global and national prevalence of diabetes in adults. Diabetes Res Clin Pract.

[26] Al-Nozha M.M., Al-Maatouq M.A., Al-Mazrou Y.Y., Al-Harthi S.S., Arafah M.R., Khalil M.Z. (2004). Diabetes mellitus in Saudi Arabia. Saudi Med J.

[27] Asfour M.G., Lambourne A., Soliman A., Al-Behlani S., Al-Asfoor D., Bold A. (1995). High prevalence of diabetes mellitus and impaired glucose tolerance in the sultanate of Oman: results of the 1991 National Survey. Diabet Med.

[28] Holman N., Forouhi N.G., Goyder E., Wild S.H. (2011). The Association of Public Health Observatories (APHO) Diabetes Prevalence Model: estimates of total diabetes prevalence for England, 2010–2030. Diabet Med.

[29] Khatib O.M.N. (2006). Guidelines for the prevention, management and care of diabetes mellitus: World Health Organization, Regional Office for the Eastern Mediterranean [EMRO Technical Publication Series, No. 32].

[30] International Diabetes and Federation (2009). Middle East and North Africa (MENA) Regional Meeting: Supporting the Implementation of the United Nations Resolution (UNR) on Diabetes (61/225) in Middle East and North Africa.

[31] Al-Nuaim A.R. (1997). Prevalence of glucose intolerance in urban and rural communities in Saudi Arabia. Diabet Med.

[32] El-Hazmi M.A.F., Warsy A.S., Al-Swailem A.R., Al-Swailem A.M., Sulaimani R. (1998). Diabetes mellitus as a health problem in Saudi Arabia. East Mediterr Health J.

[33] El-Hazmi M.A.F., Al-Swailem A., Warsy A.S., Al-Sudairy F., Sulaimani R., Al-Swailem A. (1995). The prevalence of diabetes mellitus and impaired glucose tolerance in the population of Riyadh. Ann Saudi Med.

